# Task Performance Modulates Functional Connectivity Involving the Dorsolateral Prefrontal Cortex in Patients with Schizophrenia

**DOI:** 10.3389/fpsyg.2017.00056

**Published:** 2017-02-27

**Authors:** Shihao Wu, Huiling Wang, Cheng Chen, Jilin Zou, Huan Huang, Peifu Li, Yilin Zhao, Qizhong Xu, Liang Zhang, Hesheng Wang, Sanjib Pandit, Subodh Dahal, Jun Chen, Yuan Zhou, Tianzi Jiang, Gaohua Wang

**Affiliations:** ^1^Department of Psychiatry, Renmin Hospital of Wuhan UniversityWuhan, China; ^2^Hubei Provincial Key Laboratory of Developmentally Originated DiseaseWuhan, China; ^3^Department of Radiology, Renmin Hospital of Wuhan UniversityWuhan, China; ^4^CAS Key Laboratory of Behavioral Science, Institute of PsychologyBeijing, China; ^5^Department of Psychology, University of Chinese Academy of SciencesBeijing, China; ^6^Brainnetome Center, Institute of Automatuon, Chinese Academy of SciencesBeijing, China; ^7^Key Laboratory for NeuroInformation of the Ministry of Education, School of Life Science and Technology of ChinaChengdu, China; ^8^Hubei Institute of Neurology and Psychiatry ResearchWuhan, China; ^9^Hubei University of Science and TechnologyXianning, China

**Keywords:** schizophrenia, N-back, fMRI, psychophysiological interaction (PPI), middle frontal gyrus, fusiform gyrus, inferior parietal lobule (IPL)

## Abstract

Previous studies have suggested that patients with schizophrenia and healthy controls exhibit differential activation of and connectivity involving the dorsolateral prefrontal cortex (DLPFC) during working memory tasks, though their findings remain inconsistent. The functional integration perspective further suggests that working memory performance also modulates differences in functional interactions of the DLPFC between patients and controls. To explore this possibility, 45 healthy controls and 45 patients with schizophrenia were recruited to perform a 2-back task during functional magnetic resonance imaging (fMRI). Each group was further divided into two subgroups based on task performance to examine the modulatory effect of performance on functional interactions of the DLPFC, as measured via psychophysiological interaction (PPI) analyses. We observed that, in patients with schizophrenia who exhibited impaired working memory capacity and decreased brain activation/deactivation, functional interactions between the right/left DLPFC and angular cortex were decreased relative to those of healthy controls. Furthermore, we observed an interaction effect of working memory performance and diagnosis on functional connectivity between the right/left DLPFC seed region and posterior regions such as the angular cortex, fusiform gyrus, and middle occipital gyrus. This interaction effect was mainly driven by the negative correlation between functional connectivity and performance in healthy controls, and by the positive correlation in patients with schizophrenia. These results demonstrate the effects of inter-individual differences in working memory performance on functional interactions between the DLPFC and posterior regions in patients with schizophrenia as well as healthy controls, which may shed new light on the neural basis of working memory.

## Introduction

Schizophrenia is a neuropsychiatric condition most commonly characterized by a loss of touch with reality, abnormal behavior, and impaired cognitive function. The disorder is often described in terms of positive symptoms, such as hallucinations and delusions, and negative symptoms, such as reduced emotional expression and lack of motivation (Bozikas and Andreou, 2011). In addition, recent researches have suggested that working memory dysfunction may be a core component of schizophrenia (Lee and Park, 2005; Vu et al., 2013; Cacciotti-Saija et al., 2015; Jiang et al., 2015; Mourik et al., 2015; Schwarz et al., 2016), and that such dysfunction may be the result of abnormal activity in a specific brain network (Glahn et al., 2005; Minzenberg et al., 2009; Jiang and Zhou, 2012) involving the dorsolateral prefrontal cortex (DLPFC) (Goldman-Rakic, 1999). However, results regarding the contribution of this region remain inconsistent. For example, researchers have reported a mixture of abnormally increased, decreased, or unchanged activation in the DLPFC during working memory tasks in patients with schizophrenia when compared to healthy controls (Callicott et al., 2000; Barch et al., 2002; Manoach, 2003; Driesen et al., 2008; Dauvermann et al., 2014). One meta-analysis has suggested that differences in working memory performance may account for inconsistent findings regarding activation in the DLPFC (Van Snellenberg et al., 2006). Given that working memory involves the cooperation of multiple brain regions, task performance may also influence functional interactions involving the DLPFC.

Previous studies utilizing functional connectivity analyses have reported decreased functional coupling between the DLPFC and inferior parietal lobe during working memory tasks in patients with schizophrenia (Tan et al., 2006; Rasetti et al., 2011). Furthermore, impaired frontoparietal connectivity has been linked with poor working memory performance (Tan et al., 2006). Additional studies have indicated that functional interactions between the DLPFC and other brain regions are modulated by working memory load and task difficulty, and that patients with schizophrenia exhibit deficits in such modulatory activity (Meyer-Lindenberg et al., 2005; Anticevic et al., 2012). Given that working memory performance under difficult or high-load conditions is significantly impaired in patients with schizophrenia, these findings suggest that task performance may influence functional interactions among brain regions in schizophrenia. Several studies have analyzed differences in working memory performance in order to elucidate how prefrontal organization is altered in schizophrenia, (Tan et al., 2006; Deserno et al., 2012); however, these studies focused only on task-induced activity rather than functional connectivity. Therefore, investigation of the effect of working-memory task performance on functional interactions related to the DLPFC in schizophrenia is necessary in order to understand the neural basis of working memory dysfunction in this patient population.

In the present study, we aimed to investigate the mechanism by which abnormal functional activity involving the DLPFC is modulated by working memory task performance in patients with schizophrenia. We divided both patients and controls into “high performance” and “low performance” subgroups based on the median performance score in each group. We further utilized psychophysiological interaction (PPI) analysis (Friston et al., 1997), which is commonly used in studies of schizophrenia (Boksman et al., 2005; Postma et al., 2006; Fakra et al., 2008), in order to determine the influence of activity in each brain region on functional networks involving the DLPFC. We speculated that patients with schizophrenia would exhibit altered functional connectivity among the DLPFC and multiple regions involved in working memory, and that functional interactions involving the DLPFC would be differentially modulated by task performance in patients and healthy controls.

## Materials and methods

### Participants

The study was approved by the Ethics Committee of Renmin Hospital of Wuhan University. Written informed consent forms were obtained from all participants and at least one first-degree relative of each patient prior to their participation in the study. Patients diagnosed with schizophrenia according to criteria outlined in the Diagnostic and Statistical Manual of Mental Disorders, 4th edition (DSM-IV) were recruited from the Department of Psychiatry at Renmin Hospital of Wuhan University (Wuhan, China). Upon primary diagnosis, researchers conducted a Structured Clinical Interview for DSM-IV Disorders (SCID) to confirm the diagnosis. In addition to the SCID interview, patients were evaluated by researchers who had received training in utilizing the Positive and Negative Syndrome Scale (PANSS), and patients with a PANSS score of 60 or above were recruited for the present study (*n* = 45, PANSS score: 84.20 ± 9.78). Patients were also required to meet the following inclusion criteria: (1) 18–45 years of age, (2) at least 9 years of education, (3) right-handed, and (4) Han Chinese. Exclusion criteria included diagnosis of any other DSM Axis-I disorders, severe physical illness including cardiovascular disease, electroconvulsive therapy 6 months prior to recruitment, or the presence of structural changes in the brain (such as white matter lesions), as diagnosed by a radiologist. The healthy controls were screened for personal and family history of illness using the SCID and had the same inclusion and exclusion criteria, except that healthy controls were excluded if they or their first-relatives met any diagnosis of a psychiatric disorder according to DSM-IV criteria.

### N-back task and experimental procedure

A block design, numeric n-back task (Salomon et al., 2011; Deserno et al., 2012) was used in the present study. The paradigm alternated between rest and task conditions. During rest conditions, participants were instructed to fixate on a cross at the center of the screen for the duration of five scans (i.e., 10 s). The task consisted of two conditions: a 0-back (baseline) condition and a 2-back (working memory) condition. During the task, numbers from 0 to 9 were presented on the screen. Participants were instructed to match the current letter to the number 9 during the 0-back trials and to match the current letter to the number presented two trials earlier during the 2-back trials. Before each condition, a visual cue lasting 2 s indicated the nature of the subsequent block to be presented. Each block comprised 12 stimuli, three of which were targets, each presented for 1000 ms with a 1000 ms inter-stimulus interval. In total, the task was composed of six 0-back and six 2-back blocks appearing in a semi-random order (0-2-0-0-2-2-2-0-0-2-2-0) for each participant. Throughout the task, a total of 18 targets and 54 non-targets were displayed in both the 0-back and 2-back conditions.

In order to ensure that all participants correctly understood the demands of the task, a full training session was conducted prior to scanning. Training was conducted in a quiet room. The training task was similar to the formal task, though only one 0-back and one 2-back trial were included. Accuracy was displayed on the monitor at the end of the practice task, and patients were provided with further practice opportunities when deemed necessary by the experimenter. Prior to scanning, the task instructions were presented again, and participants were required to press the appropriate response buttons. Responses were displayed to researchers as blinking lights on a Visual and Audio Stimulation System for fMRI, SA-9900 (Shenzhen Sinorad Medical Electronics Inc., Shenzhen, China) in the control room. When any participant was unable to operate the response buttons or follow the directions correctly, the experimenter explained the directions over the speaker in the control room.

### Image acquisition

Functional MRI data were acquired in the Radiology Department of Renmin Hospital of Wuhan University using a General Electric HDxt 3.0 T Scanner with an eight-channel head coil. All scans were performed by experienced radiologists. High-resolution structural images were acquired using a 3D Bravo T1-weighted sequence (repetition time = 7.8 ms; echo time = 3.0 ms; flip angle = 7°; matrix = 256 × 256; field of view = 220 × 220 mm; slice thickness = 1 mm) composed of 188 slices in a sagittal orientation. Whole-brain functional scans were collected in 32 axial slices using an echo-planar imaging (EPI) sequence (time points = 228, repetition time = 2000 ms, echo time = 30 ms; flip angle = 90°; matrix = 64 × 64; field of view = 220 × 220 mm; slice thickness = 4 mm; slice gap = 0.6 mm; voxel size = 3.8 × 3.8 × 4.0 mm). Participants were instructed to focus and respond correctly to stimuli as soon as they were presented for a total of 456 s.

### Image preprocessing

Statistical Parametric Mapping (SPM12, http://www.fil.ion.ucl.ac.uk/spm/) and Data Processing Assistant for Resting-State fMRI (DPARSF 2.3, http://www.restfmri.net) were used for data preprocessing. Prior to preprocessing, the first six volumes (no stimuli were presented during this period) were discarded to allow for signal stabilization. The remaining volumes acquired from each participant were corrected for differences in slice acquisition times. The resultant images were then realigned to correct for small movements that occurred between scans. Individual T1-weighted structural images were co-registered to the mean of the realigned EPI images. The transformed structural images were then segmented into gray matter (GM), white matter, and cerebrospinal fluid using DARTEL. The resulting maps were then registered into the Montreal Neurological Institute atlas space with an EPI template using DARTEL, and resampled to 3-mm isotropic voxels. A 6-mm full-width half-maximum Gaussian kernel was used for spatial smoothing. Images exhibiting less than 3 mm maximum displacement in x, y, or z, and less than 3°angular rotation about each axis were used in following analyses.

### Behavioral data analysis

We used the sensitivity index (*d*′) of signal detection to asses working memory performance (Wickens, 2002). The raw data of the 0-back and 2-back tasks were transformed into Z-scores in reference to Green and Swets (1966). For statistical transformation, a 0% performance was corrected to [(0 + 1)/2n] × 100%, while a 100% performance was corrected to [1 − (1/2n)] × 100%, where *n* represents the number of targets or non-targets in respect to hit rate (HR) or false rate (FR) calculation. Subtracting Z(FR) from Z(HR) provides a *d*′ score that represents the degree of goal-orientation in participant response. Participants with positive *d*′ values were considered to have provided non-random responses and were included in the subsequent analyses. Each group was then divided into two subgroups based on performance during 2-back conditions (*d2*′ score). Participants who scored above average were assigned to the “high performance” subgroup, while those who scored below average were assigned to the “low performance” group. Behavioral data analysis was conducted using SPSS 19.0 (IBM Inc., New York, USA).

### General linear model analysis

A general linear model (GLM) analysis was applied as a first-level analysis for detecting brain activation during the experimental task using SPM12. In the first-level analysis, two variables capturing the task conditions (0-back and 2-back) were used as regressors of interest. The data for the instruction times for the task blocks (cues) were regressed out as nuisance variables in the GLM. In order to account for the variance related to head motion and other spurious or regionally nonspecific variance, the following nuisance variables were included in the model: (i) 24 parameters (including six head motion parameters, six head motion parameters one time point before, and the 12 corresponding squared items) obtained by rigid body head motion correction; and (ii) five principal components from an anatomically defined noise volume of interest (VOI) (composed of white matter and cerebrospinal fluid), an approach which has been shown to accurately describe physiological noise in gray matter (Behzadi et al., 2007). We then generated individual statistical probability maps for each task (0-back or 2-back condition), as well as for task-evoked activation (2-back > 0-back) and task-evoked deactivation (2-back < 0-back).

In the second level analysis, the contrast maps (2-back > 0-back) of 45 patients and 45 controls were entered into an analysis of variance (ANOVA), with diagnosis (patients vs. controls) and 2-back performance (high vs. low) as grouping variables. Age, sex, and education were regarded as covariates of no interest. In order to further exclude the influence of head motion, the mean framewise displacement (FD) was included as a covariate in the group-level analysis (Power et al., 2012). In addition, one-sample *t*-tests were used to identify regions of task-evoked activation and deactivation across all participants. These regions were used as a mask to separate activated regions from deactivated regions in the ANOVA. The statistical significance was determined by Monte Carlo simulations to obtain a *p*-value of < 0.05 after correcting for whole brain comparisons using the AlphaSim program in the REST 1.8 software (http://www.restfmri.net). The corrected threshold corresponds to an uncorrected *p*-value of < 0.005, a cluster size dependent on the simulation based on the observed smoothness of the data. To further assess the significance of the interaction effect, the effect values in clusters of interest were extracted and averaged from individual maps for *post hoc* analysis. These data were analyzed using SPSS Version 19.0.

### Psychophysiological interaction (PPI) analysis

In order to perform context-specific analysis for connectivity, psychophysiological interaction (PPI) analyses were conducted (Friston et al., 1997). PPI can be used to determine the presence of reactivity or coactivity that is task specific in relation to a selected region. As we were interested in connectivity related to the dorsolateral prefrontal cortex (DLPFC), we selected our volumes of interest (VOIs) based on the regions showing overlapping activation across controls and patients. A mask consisting of Brodmann Area 9 was then applied to the overlapping areas, and two VOIs were selected: right DLPFC (*x* = 39, *y* = 30, *z* = 36) and left DLPFC (*x* = −48, *y* = 18, *z* = 39). Eigenvariables from a sphere with a radius of 6 mm centered at each of the coordinates were extracted from individual contrast maps (2-back > 0-back).

For each VOI, PPI analysis was conducted using SPM12. The interaction term was generated from the VOI time series and a psychological variable reflecting the working memory effect (i.e., 2-back > 0-back contrast). The interaction term was entered as a regressor of interest, while the physiological and psychological variables were entered as covariates of no interest. Subject-specific PPI regression coefficients were estimated at the first level and were then entered into an ANOVA with diagnosis and 2-back performance as grouping variables. Age, sex, education, and mean FD were regarded as covariates of no interest. The statistical significance was determined by Monte Carlo simulations to obtain a *p*-value of < 0.05 after correcting for whole brain comparisons (*p*-uncorrected < 0.005, a cluster size dependent on the simulation based on the observed smoothness of the data). *Post hoc* analyses were then conducted for the averaged effects in the regions of interest using MODPROBE, which is capable of probing single-degree-of-freedom interactions in OLS and logistic regression analyses (Hayes and Matthes, 2009).

## Results

### Demographic and clinical characteristics

Forty-five patients and 45 healthy controls were included in the data analyses. No significant difference in sex (χ^2^ < 0.001, *df* = 1, *p* = 1.00), age (*t* = −0.084, *df* = 88, *p* = 0.933), or education (*t* = 1.618, *df* = 88, *p* = 0.933) was noted between patients and controls. Participant characteristics are listed in Table 1.

**Table 1:** **Demographic and task performance of participants**.

	***df***	**Control (*n* = 45)**	**Patient (*n* = 45)**	**Statistical value**	***p***
Sex (m/f)	1	24/21	24/21	0.400^#^	0.527
Onset	44		20.74 ± 4.32		
Course	44		38.02 ± 40.53		
Positive and negative syndrome scale (PANSS) score	43		84.20 ± 9.78		
High performance			83.88 ± 7.64	−0.252^&^	0.802
Low performance			84.63 ± 12.20		
Age	88	24.07 ± 4.83	24.16 ± 5.20	−0.084^&^	0.933
High performance		23.36 ± 4.18	24.15 ± 5.18		
Low performance		25.24 ± 5.67	24.16 ± 5.37		
Education	88	13.42 ± 1.59	12.60 ± 2.81	1.618^&^	0.109
High performance		13.39 ± 1.66	12.77 ± 3.09		
Low performance		13.47 ± 1.50	12.47 ± 2.44		
d′‘0-back	88	4.00 ± 0.30	3.94 ± 0.36	0.852^∧^	0.369
High performance		4.04 ± 0.27	4.02 ± 0.36		
Low performance		3.93 ± 0.36	3.82 ± 0.35		
d′ 2-back	88	3.18 ± 0.60	2.41 ± 0.86	7.870^∧^	<0.001^*^
High performance		3.57 ± 0.28	3.00 ± 0.44		
Low performance		2.55 ± 0.42	1.59 ± 0.57		

df, degree of freedom; Analytic method:

# Chi-Square test;

& one sample/independent sample t-test;

∧ Analysis of Variance; Significance:

* *p < 0.001*.

**Figure 1:**
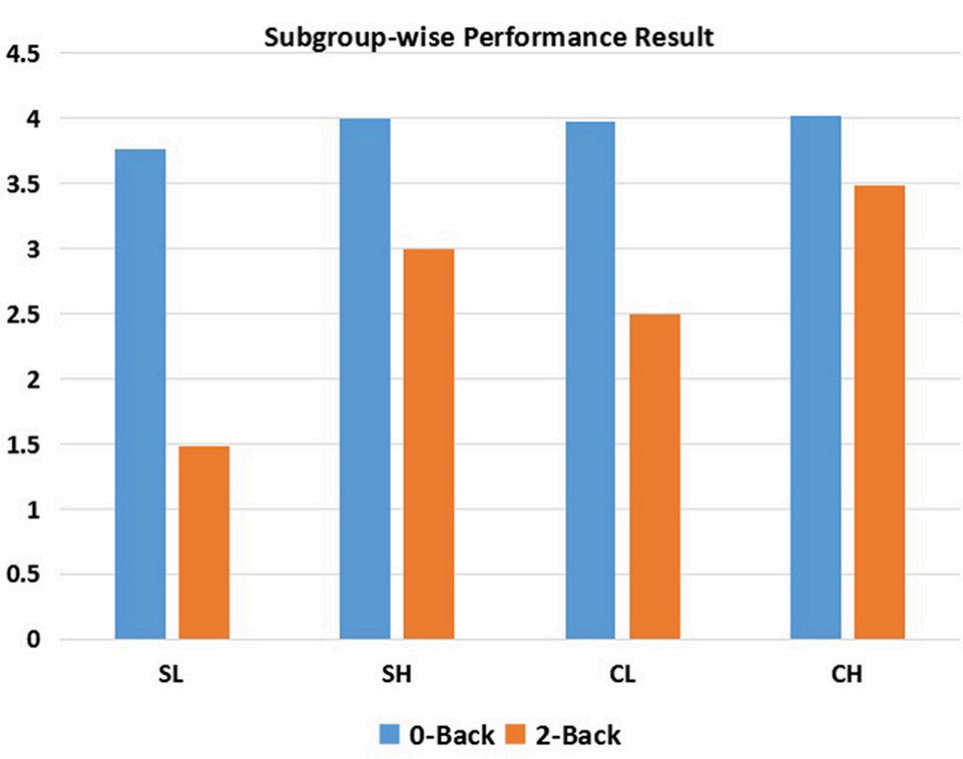
**N-back performance within each subgroup**. SL, low-performance schizophrenia group; SH, high-performance schizophrenia group; CL, low-performance control group; CH, high-performance control group.

Separate ANOVAs were conducted for 0-back and 2-back performance. For 0-back performance (*d*′0 score), neither the effect of diagnosis nor the interaction effect between diagnosis and performance was significant, while there was a significant main effect of performance (*df* = 1, *F* = 4.438, *p* = 0.038). However, significant main effects of diagnosis (*df* = 1, *F* = 68.45, *p* = < 0.001), performance (*df* = 1, *F* = 174.83, *p* < 0.001), and the interaction effect of diagnosis and performance (*df* = 1, *F* = 4.326, *p* = 0.041) were observed for 2-back performance (*d*2′ score). Zero-back and 2-back performance distributions are displayed in Figure 1.

### Brain activation

**Figure 2:**
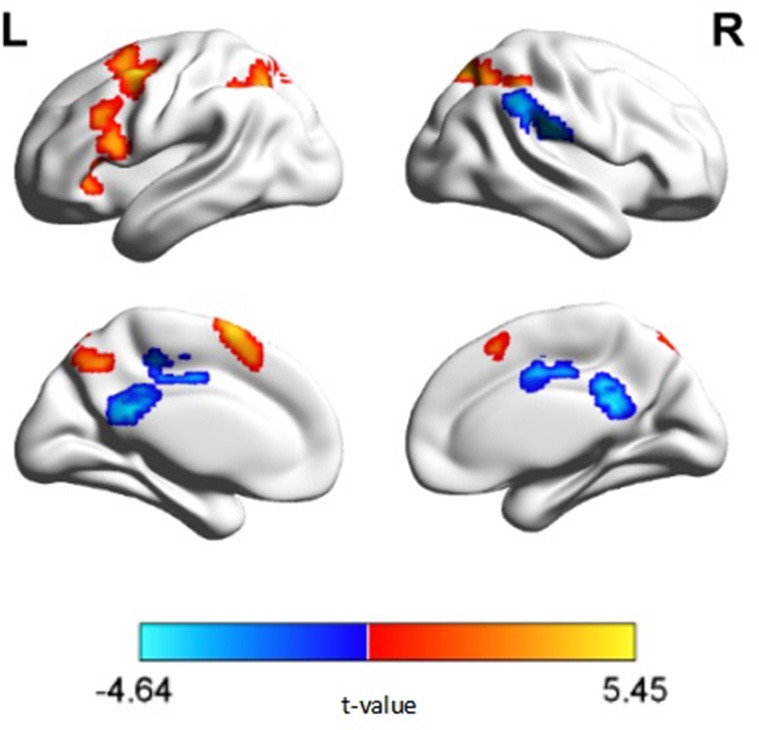
**N-back activation map of between group comparisons (***p*** < 0.05, corrected, cluster size = 108)**. Warm colors represent regions exhibiting decreased activation, while cold colors represent regions exhibiting decreased deactivation, in patients with schizophrenia relative to healthy controls.

The ANOVA revealed significant main effects of diagnosis in the right lingual gyrus, right superior parietal lobule, right supramarginal gyrus, right supra marginal gyrus, right middle cingulate gyrus, left middle frontal gyrus, left inferior parietal lobule, and left posterior cingulate. *Post hoc* analyses revealed decreased activation in the right lingual gyrus, right superior parietal lobule, left middle frontal gyrus and left inferior parietal lobule in patients with schizophrenia compared with the control group (Figure 2). Decreased deactivation was observed in the right supramarginal gyrus, right middle cingulate gyrus, and bilateral posterior cingulate cortices in patients with schizophrenia relative to those of the control group (*p* < 0.05, corrected, cluster size = 108). No interaction effect of performance × diagnosis was observed (*p* > 0.05, corrected). Peak intensity details for within- and between-group contrast are provided in Table 2.

**Table 2:** **Differences in activation according to diagnosis**.

**Hemisphere**	**Area**	**Cluster**	**Brodmann area**	**Coordinate (MNI)**			***t***
				***X***	***Y***	***Z***	
**CONTROL GROUP: ACTIVATION (CLUSTER SIZE = 183)**							
L	Sup motor area	5619	6, 9, 10	−3	−15	−51	14.39
R	Vermis	3762	18, 37	3	−45	24	8.33
L	Inferior parietal lobule	2626	7, 40	−27	−60	39	12.76
L	Caudate	258	–	−18	−3	21	7.21
**CONTROL GROUP: DEACTIVATION (CLUSTER SIZE = 183)**							
R	Mid cingulum gyrus	15822	6, 7, 18, 31	6	−15	42	−12.83
**SCHIZOPHRENIA GROUP: ACTIVATION CLUSTER SIZE = 176**							
R	Insula	4704	6, 8, 9, 10	33	24	3	10.76
R	Inferior parietal lobule	2486	7, 40	36	−54	39	9.31
L	Cerebellum crus	398	–	−39	−60	−33	6.10
L	Pyramis	198	–	−3	−84	−36	5.13
L	Midbrain	186	–	−3	−30	−18	5.07
**SCHIZOPHRENIA GROUP: DEACTIVATION CLUSTER SIZE = 176**							
R	Medial cingulum	16,719	3, 13, 18, 21, 31	3	−18	45	−9.66
R	Superior frontal gyrus	230	19	54	−69	6	−5.42
**MAIN EFFECT OF DIAGNOSTIC GROUP (ACTIVATION): CONTROL > SCHIZOPHRENIA (CLUSTER SIZE = 108)**							
R	Lingual	827	–	3	−45	−24	5.01
R	Superior parietal lobule	181	7	18	−72	57	4.03
L	Middle frontal gyrus	550	6, 9, 44	−39	3	54	5.45
L	Inferior parietal lobule	232	7, 40	−27	−57	42	4.25
**MAIN EFFECT OF DIAGNOSTIC GROUP (DEACTIVATION): CONTROL > SCHIZOPHRENIA (CLUSTER SIZE = 108)**							
R	Supra marginal gyrus	178	40	51	−27	24	−4.22
L	Posterior cingulate	147	23, 31	0	−42	21	−4.64
R	Middle cingulate gyrus	131	24, 31	6	−3	39	−3.61

*DLPFC, dorsolateral prefrontal cortex; MNI, Montreal Neurological Institute*.

### Psychophysiological interaction of the right DLPFC

**Figure 3:**
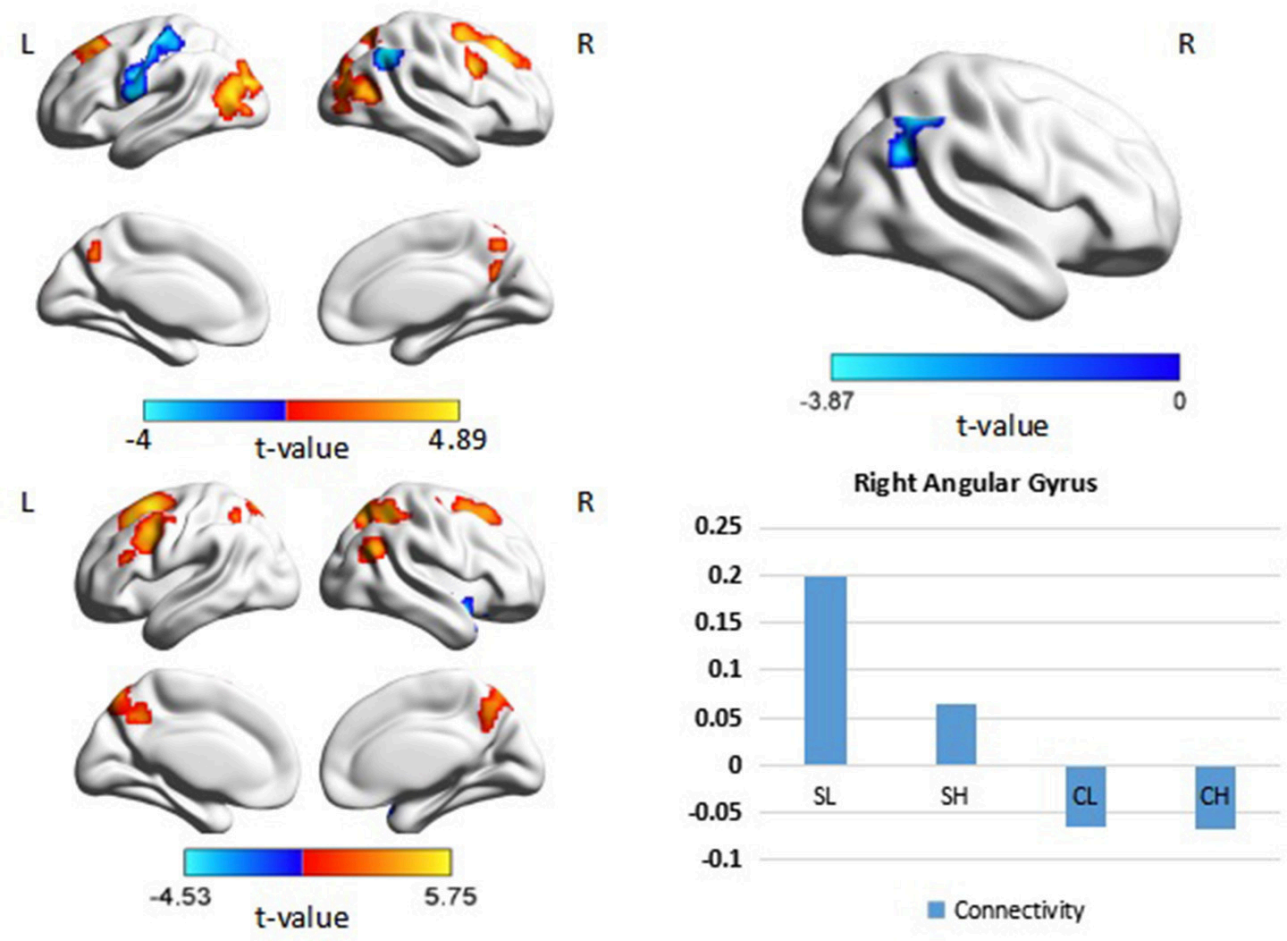
**Psychophysiological interaction effect using the right DLPFC as a seed region in each group and between group effects (***p*** < 0.05, corrected, cluster size = 69)**. Color bars indicate the t-statistic. The right bottom panel indicates the activation pattern of each subgroup. SL, low-performance schizophrenia group; SH, high-performance schizophrenia group; CL, low-performance control group; CH, high-performance control group.

When the right DLPFC was used as a seed, we observed significant connectivity between this region and the bilateral middle frontal gyri, parietal regions, and occipital regions in the control group. A similar pattern of connectivity was observed in patients with schizophrenia, with a wider area of positive connectivity with the seed in frontal regions. An ANOVA revealed significant effects of diagnosis in the right angular gyrus (*p* < 0.05 corrected, cluster size = 69). *Post hoc* analysis revealed increased connectivity between the right DLPFC and the right angular gyrus in patients with schizophrenia (Figure 3, Table 3). There was no significant main effect of performance.

**Table 3:** **Psychophysiological interaction analysis of the right DLPFC**.

**Hemisphere**	**Area**	**Cluster size**	**Broadmann area**	**Peak coordinate (MNI)**			***t***
				***x***	***y***	***z***	
**R**	**Middle frontal gyrus**	**Seed**	**9**	**39**	**30**	**36**	−
**PSYCHOPHYSIOLOGICAL INTERACTION: CONTROL, POSITIVE CONNECTIVITY (CLUSTER SIZE = 96)**							
L	Cuneus	505	19 39	−48	−78	15	4.89
R	Precuneus	449	7, 19, 39	51	−75	21	4.37
R	Superior frontal gyrus	200	8	24	27	45	4.77
R	Frontal Inferior operculum	124	9	45	9	33	4.38
R	Precuneus	118	7	3	−63	27	3.67
L	Superior frontal gyrus	116	8	−21	27	36	3.68
**PSYCHOPHYSIOLOGICAL INTERACTION: CONTROL, NEGATIVE CONNECTIVITY (CLUSTER SIZE = 96)**							
L	Rolandic operculum	399	3, 4, 6	−63	−3	12	−4.00
R	Angular gyrus	138	40	51	−54	42	−3.91
**PSYCHOPHYSIOLOGICAL INTERACTION: SCHIZOPHRENIA, POSITIVE CONNECTIVITY (CLUSTER SIZE = 94)**							
R	Precuneus	593	7 40	21	−63	54	4.92
L	Superior frontal gyrus	294	6, 8	−21	9	54	5.75
L	Precentral gyrus	269	9	−48	9	39	4.99
R	Superior frontal gyrus	202	6 8	27	3	66	3.97
R	Angular gyrus	110	39	48	−63	27	4.14
**PSYCHOPHYSIOLOGICAL INTERACTION: SCHIZOPHRENIA, NEGATIVE CONNECTIVITY (CLUSTER SIZE = 94)**							
R	Superior temporal gyrus	107	38	39	6	−18	−4.53
**PSYCHOPHYSIOLOGICAL INTERACTION: MAIN EFFECT: CONTROL**< **PATIENTS (CLUSTER SIZE = 69)**							
R	Angular gyrus	162	40	42	−60	60	−3.87
**PSYCHOPHYSIOLOGICAL INTERACTION: INTERACTIVE EFFECT: PERFORMANCE** × **DIAGNOSIS (CLUSTER SIZE = 70)**							
R	Inferior parietal lobule	124	40	51	−33	51	3.49
L	Fusiform gyrus	189	18	−33	−78	−9	−4.41
L	Middle occipital gyrus	78	19	−24	−96	15	−4.68

*All results are alpha sim corrected at p < 0.005; DLPFC, dorsolateral prefrontal cortex; MNI, Montreal Neurological Institute*.

**Figure 4:**
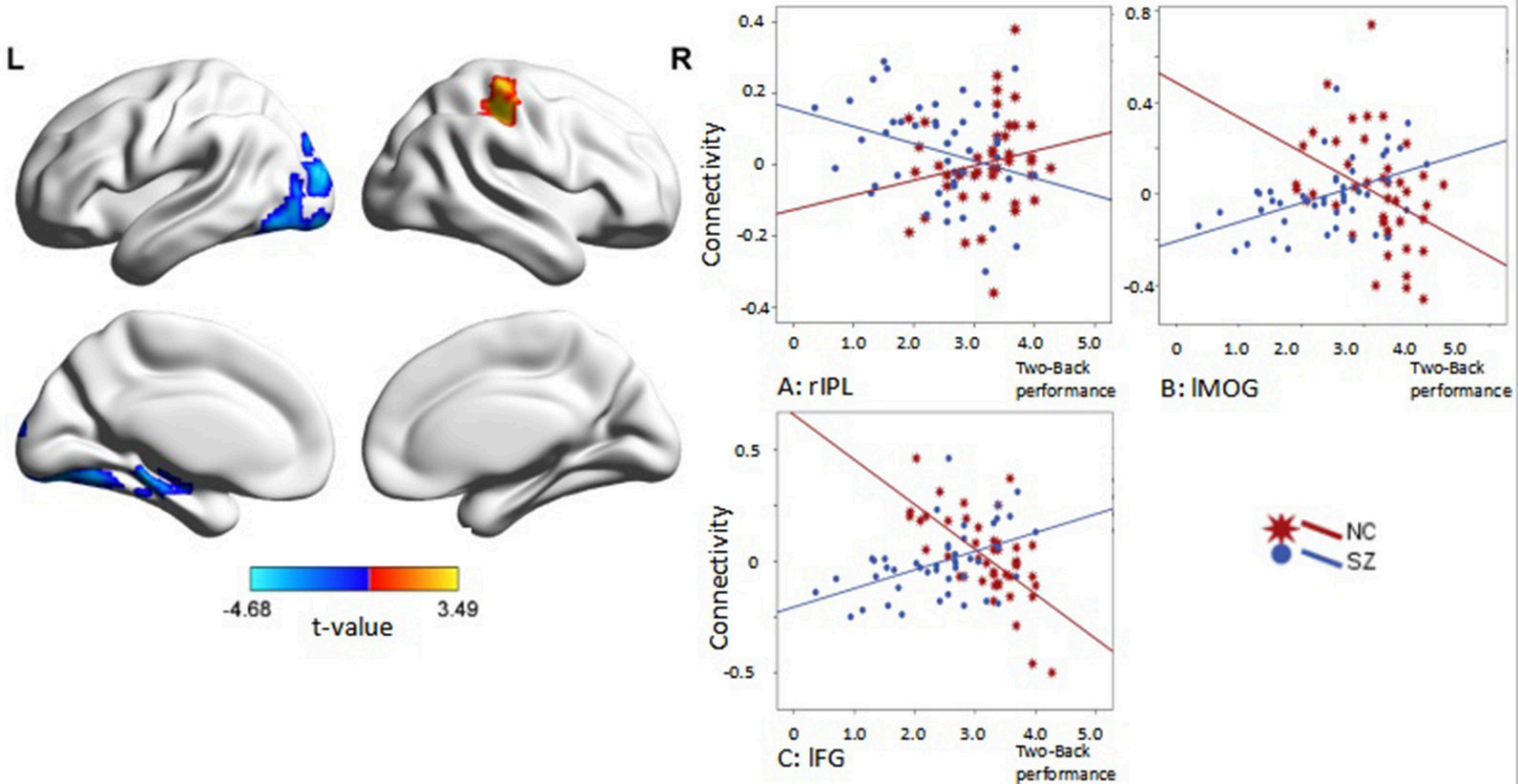
**Interaction effect of diagnosis × task performance in psychophysiological interaction analysis using the right DLPFC as the seed region (***p*** < 0.05, corrected, cluster size = 70)**. Fitted lines between n-back score and connectivity within each group were plotted. rIPL, right inferior parietal lobule; lMOG, left middle occipital lobule; lFG, left fusiform gyrus.

Interaction effects of diagnosis × performance were observed in the left fusiform gyrus, left middle occipital gyrus, and right inferior parietal lobule (IPL) (*p* < 0.05, corrected, cluster size = 70) (Figure 4, Table 3). Independent analyses of the averaged strength of functional interaction in each cluster validated the interaction effects (for the left fusiform gyrus (β = 0.28, *p* < 0.001), for the left middle occipital gyrus (β = 0.26, *p* < 0.001), for the right angular gyrus (β = −0.09, *p* = 0.03). *Post-hoc* tests of these effects showed that the functional interaction strength in the left fusiform gyrus (β = −0.20, *SE* = 0.04, *t* = −5.66, *p* < 0.001) and the left middle occipital gyrus (β = −0.15, *SE* = 0.05, *t* = −2.81, *p* = 0.006) were negatively correlated with the working memory performance measured by *d2*′ in the healthy controls, but positively correlated with performance in the patients with schizophrenia [for the left fusiform: β = 0.08, *SE* = 0.02, *t* = 3.34, *p* = 0.001; for the left middle occipital gyrus (β = 0.1, *SE* = 0.04, *t* = 2.73, *p* = 0.008)]. The functional interaction strength in the right angular gyruswas not correlated with the performance in the normal controls (β = −0.04, *SE* = 0.03, *t* = 1.27, *p* = 0.21), but negatively correlated with performance in the patients with schizophrenia (β = −0.05, *SE* = 0.02, *t* = −2.11, *p* = 0.04).

### PPI of the left DLPFC

**Figure 5:**
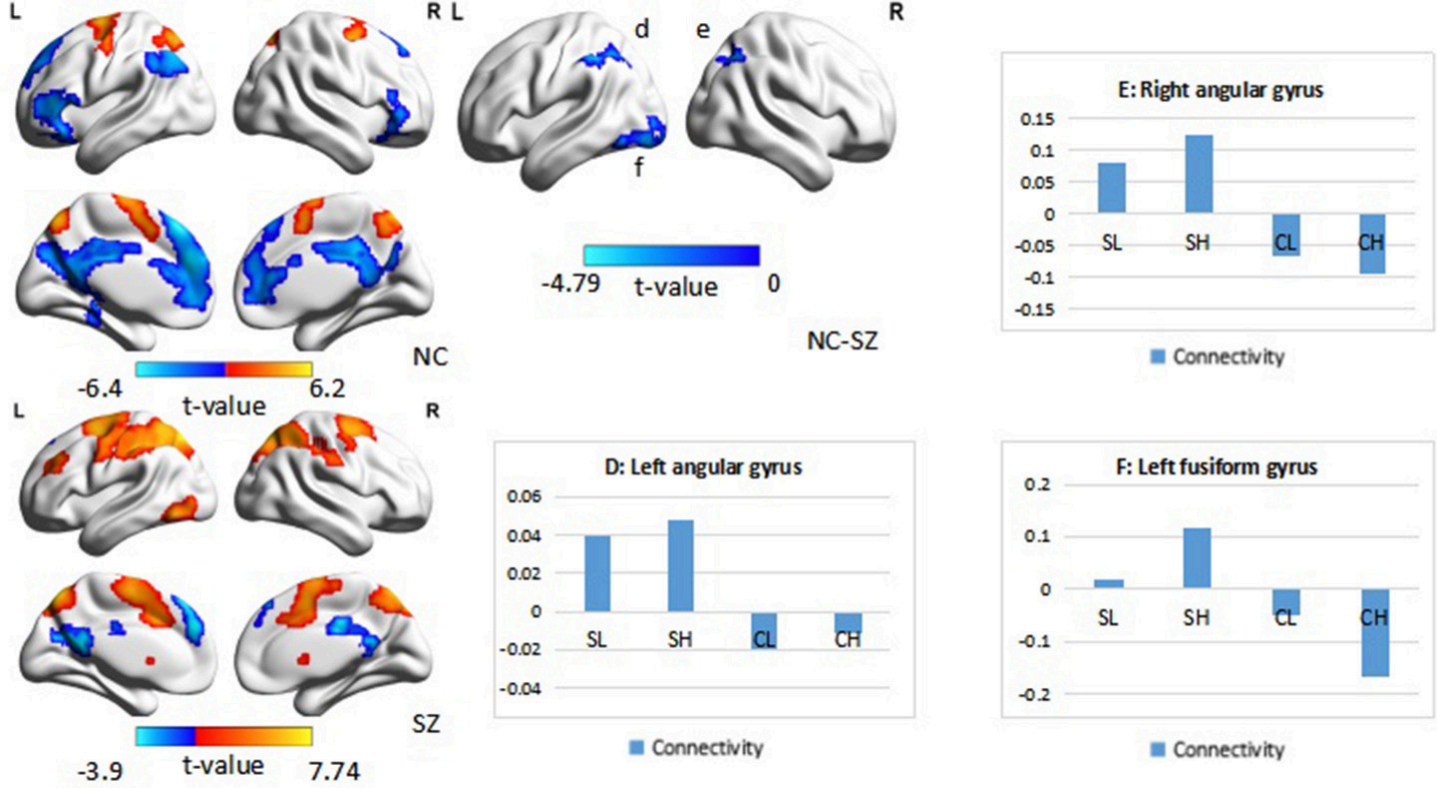
**Psychophysiological interaction effect using the left DLPFC as the seed region in each group and between group effects (***p*** < 0.05, corrected, cluster size = 86)**. The color bar represents the *t*-values. Averaged connectivity strength for each region in each subgroup is shown in the corresponding plots. SL, low-performance schizophrenia group; SH, high-performance schizophrenia group; CL, low-performance control group; CH, high-performance control group.

When the left DLPFC was used as the seed region, we observed significant positive connectivity with the seed in the bilateral supplementary motor area and bilateral superior parietal lobule, as well as significant negative connectivity in the left superior frontal gyrus, bilateral inferior frontal gyrus, left inferior parietal lobule, bilateral medial prefrontal cortex, and posterior cingulate cortex in the control group. In patients with schizophrenia, the bilateral supplementary motor area, bilateral superior parietal lobule, right putamen, right frontal middle gyrus, and left inferior temporal lobule also exhibited positive connectivity with the seed region, while the left precuneus and left superior frontal gyrus exhibited negative connectivity with the seed region. An ANOVA revealed a significant main effect of diagnosis: Patients with schizophrenia exhibited significantly greater connectivity with the seed region in the left and right angular gyrus, as well as the left fusiform gyrus (Figure 5, Table 4), relative to controls (*p* < 0.05, corrected, cluster size = 86).

**Table 4:** **Psychophysiological interaction analysis of the left DLPFC**.

**Hemisphere**	**Area**	**Cluster size**	**Broadman area**	**Peak coordinate (MNI)**			***t***
				***X***	***Y***	***Z***	
**L**	**Middle frontal gyrus**	**Seed**	**9**	−**48**	**18**	**39**	
**PSYCHOPHYSIOLOGICAL INTERACTION: CONTROL, POSITIVE CONNECTIVITY (CLUSTER SIZE = 93)**							
L	Supplementary motor area	621	6	−18	−6	66	4.92
L	Superior parietal lobule	260	7	−24	−63	54	4.89
R	Superior Parietal lobule	146	7	15	−69	57	6.20
**PSYCHOPHYSIOLOGICAL INTERACTION: CONTROL, NEGATIVE CONNECTIVITY (CLUSTER SIZE = 93)**							
L	Superior frontal gyrus	1312	8, 9, 10, 32	−3	33	51	−6.40
L	Cingulate gyrus	892	7, 23, 31	−3	−33	27	−5.65
L	Inferior frontal gyrus	369	6	−45	33	6	−5.48
L	Inferior parietal lobule	273	39, 40	−42	−66	39	−4.86
R	Orbital frontal lobe	182	11, 46, 47	30	39	−12	−4.34
**PSYCHOPHYSIOLOGICAL INTERACTION: PATIENTS, POSITIVE CONNECTIVITY (CLUSTER SIZE = 126)**							
L	Superior parietal lobule	3229	6	−15	−72	54	7.74
R	Superior parietal lobule	1002	7, 40	15	−66	57	6.64
R	Caudate	197	–	9	18	−3	3.72
L	Putamen	181	–	−12	9	−3	4.21
L	Middle frontal gyrus	173	9	−30	36	24	4.84
L	Inf. temporal lobule	133	19	−48	−66	−9	5.60
**PSYCHOPHYSIOLOGICAL INTERACTION: PATIENT, NEGATIVE CONNECTIVITY (CLUSTER SIZE = 126)**							
L	Precuneus	285	23, 31	−6	−48	15	−3.87
L	Sup. frontal gyrus	170	8, 9	−3	48	30	−3.90
**PSYCHOPHYSIOLOGICAL INTERACTION: MAIN EFFECT OF DIAGNOSTIC GROUP (CONTROL > PATIENTS)**							
–	None	–	–	–	–	–	–
**PSYCHOPHYSIOLOGICAL INTERACTION: MAIN EFFECT OF DIAGNOSTIC GROUP (CONTROL**< **PATIENTS) (CLUSTER SIZE = 86)**							
L	Angular gyrus	282	40	−51	−42	42	−3.90
R	Angular gyrus	153	7	33	−69	45	−4.78
L	Fusiform gyrus	104	18	−36	−84	−12	−3.90
**PSYCHOLOGICAL INTERACTION: MAIN EFFECT OF PERFORMANCE (BETTER**< **WORSE) (CLUSTER SIZE = 61)**							
L	Angular gyrus	99	19, 39	−39	−87	24	−3.42
**PSYCHOPHYSIOLOGICAL INTERACTION: INTERACTIVE EFFECT (PERFORMANCE** × **DIAGNOSIS) (CLUSTER SIZE = 68)**							
L	Fusiform gyrus	127	18	−33	−75	−15	−3.61

*DLPFC, dorsolateral prefrontal cortex; MNI, Montreal Neurological Institute*.

An ANOVA suggested that there was a significant performance effect in the left angular gyrus (*p* < 0.05, corrected, cluster size = 61). *Post hoc* analysis revealed significantly greater positive connectivity between the seed region and left angular gyrus in participants with low performance. Positive connectivity patterns were observed in patients with low performance overall, while significant patterns of dysconnectivity were observed in control participants.

**Figure 6:**
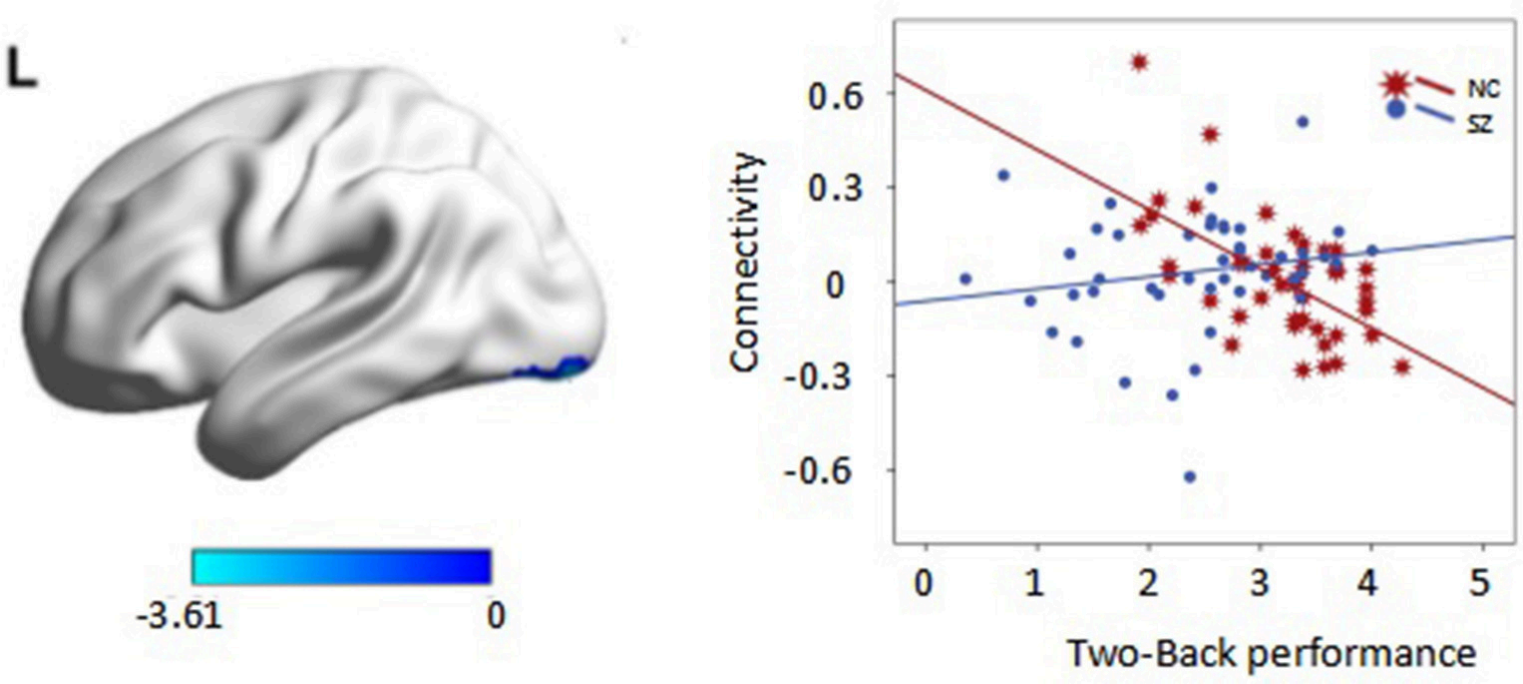
**Interaction effect of diagnosis × task performance in psychophysiological interaction analysis using the left dorsolateral prefrontal cortex (DLPFC) as the seed region (***p*** < 0.05, corrected, cluster size = 68)**. Average connectivity strength in each subgroup is shown in the plot on the right. SL, low-performance schizophrenia group; SH, high-performance schizophrenia group; CL, low-performance control group; CH, high-performance control group.

Interaction effects of diagnosis × performance were observed in the left fusiform gyrus, (*p* < 0.05, corrected, cluster size = 68) (Figure 6, Table 4). Independent analyses of the averaged strength of functional interaction in the left fusiform gyrus validated the interaction effects (β = 0.23, *p* < 0.001). *Post-hoc* test showed that the functional interaction strength in the left fusiform gyrus (β = −0.19, *SE* = 0.04, *t* = −4.40, *p* < 0.001) was negatively correlated with the working memory performance measured by *d2*′ in the normal controls, but not correlated with performance in the patients with schizophrenia (β = 0.04, *SE* = 0.03, *t* = 1.28, *p* = 0.2). Please see Table 4 for reference.

## Discussion

In the present study, we investigated the influence of inter-individual differences in working memory performance on the functional connectivity of the bilateral DLPFC in patients with schizophrenia and healthy controls. As we hypothesized, we observed an interaction effect of working memory performance and diagnosis on functional connectivity between the right/left DLPFC and angular cortex, fusiform gyrus, and middle occipital gyrus. The interaction effect was mainly driven by the negative correlation between functional connectivity and performance in healthy controls but by the positive correlation between these factors in patients with schizophrenia. The observed differential effects of inter-individual differences in working memory performance on functional interactions between the DLPFC and posterior regions in patients with schizophrenia relative to healthy controls may provide new insight into the neural basis of working memory.

### Decreased brain activation and deactivation in schizophrenia

We observed decreased activation in the left lateral prefrontal cortex and bilateral parietal cortex in patients with schizophrenia. Lateral prefrontal and posterior parietal regions are essential in working memory, and reduced activation in patients of the present study may indicate that schizophrenia is associated with impairments in the maintenance and internal representation of stimuli as well as in motor plan storage (Shulman et al., 1997; McKiernan et al., 2003; Todd et al., 2005; Torrey, 2007; Mayer et al., 2009; Sheffield et al., 2015). Previous studies have indicated that lateral PFC activity may exhibit differential responses to various task components, such as verbal information processing (Curtis et al., 1999), abstract information integration, and task difficulty in healthy controls (Nee and D'Esposito, 2016), though such increases in activity may not be observed in patients with schizophrenia (Callicott et al., 2003). These studies suggest that the PFC is selectively involved in successful working memory processing during tasks with higher executive load in healthy individuals, while our findings indicate that patients with schizophrenia exhibit significantly less activation in this region. However, Jiang et al. (2015) reported contrasting results, which indicated that patients with'. schizophrenia exhibit significantly greater activation in the frontal and parietal lobes. This increase in activation was explained as a compensatory mechanism occurring in response to inefficient brain function, which necessitates the use of additional resources in task-related regions for the completion of cognitive tasks. The parietal regions are implicated in a variety of functions, including sensorimotor integration (Fogassi et al., 2005) and task execution (Singh-Curry and Husain, 2009), and a recent study involving patients with schizophrenia experiencing acute psychotic symptoms (Yildiz et al., 2011) has suggested that excessive activation in the parietal cortex is associated with the presence of delusions of alien control, although such abnormalities tend to resolve when symptoms are controlled (Spence et al., 1997; Menon et al., 2001). These findings, in accordance with those of the present study, suggest that patients with schizophrenia exhibit alterations in activation/deactivation patterns during task performance, and that such difference may be associated with the neurological basis of cognitive impairment in this patient population.

### Group differences in the neural correlates of and functional connectivity associated with working memory performance

Previous studies have discussed the effects of task performance on task-induced activation and connectivity (Meda et al., 2009; Unschuld et al., 2014). However, other researchers have reported a lack of task-modulation and interactive effects (Kang et al., 2011). Such inconsistencies prompted us to investigate whether these factors interacted to influence connectivity between the prefrontal cortex—specifically Brodmann area (BA) 9—and other brain regions. Our results suggest that both task performance and diagnosis modulate connectivity between the DLPFC and left fusiform gyrus, bilateral angular gyrus, and left middle occipital lobule. In the present study, interactions between the right frontoparietal region and the DLPFC exhibited opposite patterns of connectivity among patients and controls: While patients exhibited positive connectivity between the DLPFC and the bilateral angular gyrus and left fusiform gyrus, controls displayed an overall negative pattern of connectivity these regions. Specifically, patients with higher performance (SH group) exhibited greater strength of connectivity, which decreased with improvements in performance in healthy controls in all regions exhibiting effective connectivity with left DLPFC. These findings suggest that, in order to obtain similar task performance, patients with schizophrenia must relocate cognitive resources, consistent with the findings of previous study (Thermenos et al., 2005). Taken together, these results provide evidence in support of the “cortical inefficiency” model (Manoach et al., 1999, 2000; Callicott et al., 2000), in which enhanced activation patterns are required in order to recruit sufficient cognitive resources to maintain task performance, particularly in the DLPFC. The middle occipital lobule plays a major role in higher-level visual perception.

Kim et al. (2010) have suggested that memorization and visual searching for a distinctive target may increase activity in the occipital region, and that such activity is correlated with the difficulty of the task. Although this task-based interpretation does not fully align with the aforementioned cognition-based interpretation, these findings further highlight the possible correlation between task performance and connectivity. However, our study differs in that our results suggest that the highest levels of activation are observed among patients of the SL group, while Kim et al. (2010) have suggested that performance is negatively correlated with activation status in patients with schizophrenia. The increased connectivity as well as the failure of deactivation observed in patients of the present study supports the notion that task completion may require greater assistance from the occipital region in these patients.

Using PPI analysis, we observed a significant interaction between task performance and diagnosis on the functional connectivity of both the left and right DLPFC. Using the right DLPFC as the seed region, we observed negative correlations between the functional interaction of this region with the left fusiform in healthy controls, although positive correlations were observed in patients with schizophrenia. Using the left DLPFC as the seed region, we obtained a similar pattern in patients with schizophrenia, although no correlation was observed for healthy controls. The fusiform gyrus is considered a key structure for functionally specialized computations associated with high-level vision, including facial perception, object recognition, and reading (Weiner and Zilles, 2016), as well as social perception and judgment (Pujol et al., 2009). We speculate that the recognition function is activated for the completion of working memory (2-back) tasks: The PFC is involved in stimulus presentation and perception, while the fusiform gyrus is involved in attention, recognition, and classification. As previous investigators have suggested, the fusiform gyrus exhibits a tendency of deactivation with increasing task difficulty (Henseler et al., 2010). However, in our study, when interconnected with the right DLPFC, such deactivation was visible in both the CH and SL groups. On the other hand, failures in deactivation were noted in the SH group, while increased connectivity was noted in the CL group, suggesting that the deactivating nature of the fusiform gyrus during working memory tasks is plausible. Relatively greater positive connectivity among patients of the SH and CH groups was also observed in other regions, with the exception of connectivity between the left DLPFC and left fusiform gyrus under the main effect of performance. In high performance groups (CH and SH), a significantly greater deactivation was observed, suggesting an influence of performance. Overall, the patterns of connectivity observed for the left and right DLPFC also resemble the cross-over model proposed by Karlsgodt et al. (2009). These results indicate that patients with schizophrenia may exhibit increased connectivity during tasks with greater cognitive demand due to cognitive relocation, and that controls may exhibit decreased connectivity during such tasks, which require decreased cognitive allocation.

We also observed increased connectivity in the right fronto-occipital region in the patient group. As previously mentioned, the “cortical inefficiency” model states that additional neurons must be recruited in patients with schizophrenia in order to maintain a sufficient level of task performance (Manoach et al., 1999, 2000; Callicott et al., 2000). Specifically, patients with average performance in the present study exhibited the highest connectivity strength, which had a tendency to decrease with increases in performance in each group. Similarly, we observed that the functional interaction between the left DLPFC and left fusiform gyrus also exhibited this pattern. These findings support the notion that, in order to achieve task performance similar to that of controls, patients with schizophrenia require cognitive resource relocation, consistent with the findings of previous studies (Thermenos et al., 2005).

In general, our findings provide evidence in support of the cross-over between-subjects model, which has been proposed to interpret the influence of inter-individual differences in working memory performance on brain activation (Karlsgodt et al., 2009), although our findings extend this model to incorporate functional connectivity of the DLPFC. Among healthy controls, low performers exhibit increased functional connectivity when compared to high performers; while, among patients, decreased performance is correlated with decreased functional interactions related to the DLPFC. Several other models have been proposed to interpret the influence of inter-individual differences in working memory performance on brain activity, such as the inverted-U model (Manoach, 2003). This model was initially proposed to interpret the influence of task load or task difficulty on DLPFC activation in both patients with schizophrenia and healthy controls. The within-group inverted-U model was then extended, and a between-group model was developed to reconcile inconsistent results in working memory studies, revealing that prefrontal activation reflects inter-individual differences in performance (Deserno et al., 2012). However, this between-group inverted-U model is inherently consistent with the cross-over between-subjects model in that both show a negative correlation between performance and DLPFC activity in healthy controls but a positive correlation in patients with schizophrenia. A multi-level model has also been proposed to explain group differences in brain activity during working memory tasks, in which the participant's behavior determines his or her position on a linear pattern observed in the cross-over between-subjects model, setting the range within which their activation will vary as task difficulty changes (Karlsgodt et al., 2009). Future studies should utilize working memory tasks of higher load (e.g., 3-back task, or Sternberg-style item recognition task) to investigate whether this model aligns with functional interactions observed for the DLPFC.

However, we also observed an exception to the cross-over between-subjects model. In the connectivity between the right DLPFC and right angular gyrus, we observed no correlation between functional interactions of the right DLPFC and working memory performance in healthy controls, although a negative correlation was observed in patients with schizophrenia. This result is interesting considering the importance of frontoparietal functional connectivity in working memory (Kim et al., 2003; Schlösser et al., 2003; Barch and Csernansky, 2007). Frontoparietal connectivity is known to be associated with attentional control (Wang et al., 2010) and optimization of memory retention (Babiloni et al., 2004). Dauvermann et al. (2014) have suggested that the results of previous studies, however, are not consistent with regard to frontoparietal activity. Meda et al. (2009) performed an independent component analysis, the results of which suggested that the brains of patients with schizophrenia may have been less engaged in the n-back task, thus resulting in weaker connectivity overall. The left fusiform gyrus has also been observed to exhibit effective connectivity with the bilateral DLPFC. Although the task was not exactly the same as that utilized by Lamp et al. (2016), both research groups have suggested that the left fusiform gyrus is involved in categorical representation and verbal rehearsal. On the other hand, Galashan et al. (2015) have suggested that the left fusiform gyrus may also be involved in target/non-target discrimination. Druzgal and D'Esposito (2001, 2003) have suggested that, although mainly implicated in the processing of visual and emotional stimuli, fusiform gyrus activation may also co-occur along with DLPFC activation during working memory tasks, especially when there is an increase in working memory load. Kang et al. (2011) proposed that the fusiform gyrus exhibits differential activation, and that the connectivity of this region with the PFC is correlated with both memory load and performance.

Dehaene et al. (2003) have noted that the angular gyrus may be involved in the verbal processing of numeric tasks (i.e., memorizing a series of numbers), while the anterior superior parietal lobule may be associated with attention to numeric stimuli. Therefore, our results also suggest that patients require co-activation in the angular gyrus for attention, identification, and discrimination of numeric stimuli, although the main function of this region may be to assist in feedback provision for decision-making. Our findings are in accordance with those of a study conducted by Thermenos et al. (2005), in which activation in the inferior parietal region appeared to be more significant in patients than controls. The findings of Zhou et al. (2014) also suggest that patients recruit additional cognitive resources, thus resulting in over-coupling of brain regions. However, our findings also suggest that failure to establish connectivity, as well as failure of inhibition or activation, also occur. These findings are in accordance with those of Repovš and Barch (2012), who observed that performance is negatively correlated with activation and connectivity. Therefore, this exception suggest that there may be other models useful for interpreting the influence of inter-individual differences in working memory performance on functional connectivity, although further studies are required.

The current study extends existing knowledge on the influence of inter-individual differences in working memory performance on brain activation, from the level of functional segregation (activation) to the level of functional integration (functional interaction measured using PPI). These observed differences in functional integration during working memory tasks between patients with schizophrenia and controls provide new insight into the possible neural basis of working memory impairment in schizophrenia. Future studies should include working memory tasks with higher cognitive demand (e.g., 3-back task, or Sternberg-style item recognition task) to further examine functional interactions of the DLPFC, and to evaluate the cortical inefficiency model with more appropriate designs.

## Author contributions

SW: Main author, also responsible for data acquisition and analysis. HLW: Co-author, project coordination in Renmin Hospital of Wuhan University; CC, JZ, HH, PL, and HSW: Patient screening, evaluation, and neuroimaging data analysis. YLZ, QX, and LZ: Neuroimaging data acquisition. JC: Radiology Department coordinator. SP, SD: Behavioral data analysis. YZ: Project coordinator in Beijing (Institute of Psychology, Chinese Academy of Sciences), majority of contribution involved supporting data analysis and editing. TJ: Project coordinator, Beijing (LIAMA Center for Computational Medicine, National Laboratory of Pattern Recognition, Institute of Automation, Chinese Academy of Sciences), aided in data acquisition and analysis. GW: Project manager. All authors have taken part in reading and providing insight for this manuscript and have agreed to the publication of this manuscript.

## Funding

This study was supported by grants from the National 973 Program of China (No. 2011CB707805), the National Natural Science Foundation of China (No. 91132301), and the Natural Science Foundation of Hubei Province (No. 2014CFB732).

### Conflict of interest statement

The authors declare that the research was conducted in the absence of any commercial or financial relationships that could be construed as a potential conflict of interest.
